# Development and international multicenter evaluation of a second-generation immunochromatography test for the serological diagnosis of melioidosis

**DOI:** 10.1371/journal.pntd.0014484

**Published:** 2026-07-06

**Authors:** Hasyanee Binmaeil, Thanakon Bunsong, Koukeo Phommasone, Matthew T. Robinson, Manivanh Vongsouvath, Elizabeth A. Ashley, Enoka Corea, Trung Thanh Trinh, Shirley Yi Fen Hii, Rohaidah Hashim, Habib Abdul Hakim Esa, Tonnii Sia, Yuwana Podin, Robert Norton, Sophie Cunningham, Jamal I-Ching Sam, Christina Yek, Ella M. Meumann, Bart J. Currie, Mary N. Burtnick, Paul J. Brett, Narisara Chantratita

**Affiliations:** 1 Department of Microbiology and Immunology, Faculty of Tropical Medicine, Mahidol University, Bangkok, Thailand; 2 Lao-Oxford-Mahosot Hospital-Wellcome Trust Research Unit, Microbiology Laboratory, Mahosot Hospital, Vientiane, Lao People’s Democratic Republic; 3 Centre for Tropical Medicine and Global Health, Nuffield Department of Medicine, University of Oxford, Oxford, United Kingdom; 4 Department of Medical Microbiology and Immunology, Faculty of Medicine, University of Colombo, Colombo, Sri Lanka; 5 VNU-Institute of Microbiology and Biotechnology, Vietnam National University, Hanoi, Vietnam; 6 Bacteriology Unit, Infectious Diseases Research Centre, Institute for Medical Research, National Institutes of Health, Selangor, Malaysia; 7 Department of Medicine, Miri Hospital, Miri, Malaysia; 8 Institute of Health and Community Medicine, Universiti Malaysia Sarawak, Sarawak, Malaysia; 9 Pathology Queensland Townsville, Townsville University Hospital, Townsville, Queensland, Australia; 10 Department of Medical Microbiology, Faculty of Medicine, Universiti Malaya, Kuala Lumpur, Malaysia; 11 International Center of Excellence in Research, U.S. National Institute of Allergy and Infectious Diseases, National Institutes of Health, Phnom Penh, Cambodia; 12 Laboratory of Clinical Immunology and Microbiology, U.S. National Institute of Allergy and Infectious Diseases, National Institutes of Health, Maryland, United States of America; 13 Global and Tropical Health Division, Menzies School of Health Research, Charles Darwin University, Darwin, Australia; 14 Department of Infectious Diseases, Royal Darwin Hospital, Darwin, Australia; 15 Microbiology Department, Territory Pathology, Darwin, Australia; 16 Department of Microbiology and Immunology, University of Nevada, Reno School of Medicine, Reno, Nevada, United States of America; 17 Mahidol-Oxford Tropical Medicine Research Unit, Faculty of Tropical Medicine, Mahidol University, Bangkok, Thailand; 18 The Academy of Science, The Royal Society of Thailand, Bangkok, Thailand; Colorado State University, UNITED STATES OF AMERICA

## Abstract

Melioidosis is a life-threatening infectious disease caused by *Burkholderia pseudomallei*. Early and accurate diagnosis is critical for timely treatment and improved outcomes. Although highly endemic in tropical regions, it remains underdiagnosed due to limitations of current diagnostic methods. Bacterial culture, the diagnostic gold standard, is time-consuming, prone to misidentified species, requires specialized laboratory facilities, and has low sensitivity. The indirect hemagglutination assay is also unreliable due to poor sensitivity and specificity. We developed a second-generation immunochromatography test (Hcp1-ICT) to detect anti-Hcp1 IgG antibodies against hemolysin co-regulated protein 1 of *B. pseudomallei* and evaluated its diagnostic performance in an international multi-center study. A total of 1,838 stored serum samples from 601 culture-proven melioidosis patients, 598 healthy individuals and 639 patients with non-melioidosis infections in Thailand, Lao PDR, Vietnam, Malaysia, Sri Lanka, Cambodia, and Australia were analyzed for diagnosis of melioidosis. The sensitivities in Thailand, Lao PDR, Vietnam, Malaysia, Sri Lanka, Cambodia, and Australia were 92%, 77%, 76%, 80%, 74%, 90%, and 48%, respectively. The specificities with healthy donor samples were 96%, 79%, 98%, 100%, 100%, 87%, and 100%, while the specificities for cases with samples from other infections other than melioidosis were 97%, 87%, 98%, 98%, 100%, 90%, and 94%, respectively. These findings indicate that the Hcp1-ICT is a promising point-of-care tool for serodiagnosis of melioidosis. However, its variable performance across regions underscores the need for prospective validation locally in various regions to optimize its diagnostic utility and facilitate implementation in both referral centers and resource-limited settings.

## Author summary

Melioidosis, caused by *B. pseudomallei*, is a frequently fatal infection with an estimated 165,000 cases and 89,000 deaths annually. Diagnosis is delayed in many tropical regions because bacterial culture, the current gold standard, is slow, requires specialized facilities, and has limited sensitivity. The widely used indirect hemagglutination assay used for serodiagnosis of melioidosis performs poorly in endemic areas due to low sensitivity and specificity. Thus, rapid, accurate, point-of-care serological tests are urgently needed to guide early treatment and reduce mortality. We previously developed a first-generation Hcp1 Immunochromatographic test (Hcp1-ICT) to detect anti-Hcp1 IgG antibodies against hemolysin co-regulated protein-1 (Hcp1), a key virulence factor of *B. pseudomallei*. Although initial results in Thailand were promising, its performance required optimization and broader validation. In this study, we report the development of a second-generation Hcp1-ICT and its first international multicenter evaluation using 1,838 serum samples from patients in seven countries. Our findings demonstrate strong diagnostic potential, reveal regional variability in performance, and support the role of the second-generation Hcp1-ICT as a rapid, simple, suitable for both referral centers and resource-limited settings.

## Introduction

Melioidosis is an infectious disease caused by *Burkholderia pseudomallei*, a Gram-negative saprophytic bacillus found in soil and surface waters, and widely distributed in tropical and subtropical regions [1]. *B. pseudomallei* can survive in extreme environmental conditions and infect both animals and humans via percutaneous inoculation, ingestion or inhalation of contaminated soil or water [1]. This bacterium is endemic to Southeast Asia, South Asia, and Northern Australia, with cases increasingly reported in Africa, the Pacific, and the Americas [1,2]. Most cases of melioidosis occur during the rainy season when heavy precipitation or flooding, occurs, creating favorable conditions for the spread of *B. pseudomallei* [1,3]. A previous study estimates that the global burden of melioidosis is 165,000 cases annually, resulting in 89,000 deaths, highlighting its significant impact on public health [4]. Despite its putative epidemiological burden, it is likely that melioidosis is frequently underdiagnosed and misdiagnosed in many tropical countries, and is currently not officially recognized by the World Health Organization (WHO) as a neglected tropical disease [2].

The clinical manifestations of melioidosis are highly variable, ranging from chronic infections that mimic other illnesses to acute pneumonias and septicemia, which can complicate early clinical diagnosis [5,6]. A prospective study of melioidosis patients from northeastern Thailand, a highly endemic region with a mortality rate of 40%, reported that lung infections were the most common presentation (42%) whereas skin and soft tissue infections were less frequent (23%) [7]. In Australia, pneumonia remains the predominant presentation, with bacteremia reported in 55–74% of cases, particularly in Far North Queensland [8,9]. Genitourinary involvement is frequent with prostatic abscesses in up to 21% of males [10], osteomyelitis and septic arthritis occur in up to 16% [9], and neurological disease affects approximately 5% of patients [11]. Acute melioidosis can present as severe pneumonia, or systemic sepsis, and can progress to multi-organ failure, with a fatality rate of approximately 50% among septicemic cases [1,12]. Chronic melioidosis, a low-grade febrile illness, is frequently misdiagnosed as TB, with up to 10% of suspected chronic TB cases in endemic regions being undiagnosed melioidosis [4,13]. With case fatality rates of 90% in untreated cases, melioidosis should be suspected in febrile patients from endemic areas, particularly those with, pneumonia, abscesses, sepsis or diabetes-related infections that is unresponsive to standard antibiotics [1,4,14]. Early treatment with ceftazidime or meropenem is critical; however, delays in diagnosis, partly due to limited access to rapid diagnostic tests and bacterial culture, remain a major challenge in resource-limited settings [2,6].

The gold standard for diagnosis of melioidosis is culture of *B. pseudomallei*. However, the slow turnaround time (2–7 days) for culture can delay treatment and increase mortality [1,4,15]. Bacteria-positive culture sites may be difficult to access because they require specialized clinical training, techniques, or equipment, involve high-risk procedures (e.g., sampling from the lower respiratory tract or central nervous system), or may be unknown in cases of cryptic abscesses. Diagnostic accuracy is further limited by the technical complexity of culture, which requires trained personnel and, in some jurisdictions, specialized laboratory facilities. In resource-limited settings, *B. pseudomallei* is frequently misidentified as *Pseudomonas* species, leading to inappropriate treatment [16]. Inadequate laboratory infrastructure in endemic regions contributes to delayed diagnosis, underreporting, and misdiagnosis [1,14].

Serological tests such as the indirect hemagglutination assay (IHA) are widely used to detect *B. pseudomallei* infections but have limited diagnostic accuracy, with reported sensitivity and specificity of approximately 60% in endemic regions [4,15,17–19]. Given these limitations, rapid, reliable point-of-care (POC) diagnostics that have high sensitivity and specificity are urgently needed to facilitate early detection and timely treatment, especially in low-resource settings [1,2,14]. An antigen-based assay, the InBios Active Melioidosis Detect (AMD) Rapid Diagnostic Test*,* has recently been developed to detect *B. pseudomallei* capsular polysaccharide antigen in clinical specimens [20]. This assay enables direct pathogen detection and may be particularly useful in acute infection. However, its reported sensitivity varies depending on specimen type and bacterial load, and it is currently available for research use only (RUO).

To address this need, we recently developed an immunochromatographic test (ICT) targeting hemolysin co-regulated protein 1 (Hcp1), a component of the virulence-associated type VI secretion system (T6SS) that is essential for the intracellular behavior of *B. pseudomallei* [21]. Hcp1 is highly immunogenic during human infection and elicits strong antibody responses in patients with melioidosis [21–23]. In addition, Hcp1 can bind to the surface of host antigen-presenting cells, potentially enhancing its immunogenicity [24]. The high antibody titers observed against Hcp1 and its improved serodiagnostic performance in endemic settings support its suitability as a target antigen for the development of rapid point-of-care diagnostic tests [17,25,26]. This Hcp1-ICT detects circulating anti-Hcp1 IgG antibodies to Hcp1 using a single drop of blood or serum, provides results within 15 minutes, and does not require specialized laboratory infrastructure. Initial evaluations of this first-generation Hcp1-ICT demonstrated promising performance, with 88.3% sensitivity and 86.1% specificity in serum samples from Thailand [17]. A prospective study with suspected melioidosis patients using whole blood reported 74.5% sensitivity and 79.7% specificity, underscoring its potential for detecting culture-negative cases, particularly those suppressed by prior antibiotic use [27].

In this study, we developed a second-generation Hcp1-ICT with improved performance for use with serum, plasma, and whole blood. We conducted an international multicenter evaluation of the second-generation Hcp1-ICT using 1,838 serum samples from seven countries. Our objectives were to manufacture the second-generation Hcp1-ICT at scale and to evaluate the diagnostic performance of this POC test across different populations.

## Materials and methods

### Ethical approval

For the initial evaluation, stored serum, plasma, and whole blood samples were collected from previous prospective studies conducted between 2021 and 2023. These studies were reviewed and approved by the Ethics Committee of the Faculty of Tropical Medicine, Mahidol University (MUTM 2021-050-01 and MUTM 2022-020-01). These studies were also approved by the U.S. Army Medical Research and Development Command, Office of Human Research Oversight E03049.1a. Written informed consent was obtained from all participants.

For the international evaluation, stored serum samples were obtained from the respective study sites: Thailand (MUTM 2021-050-01), Lao PDR (N. 968/REC, and 922/REC), Vietnam (04/2020/CN-HDDD), Universiti Malaya Medical Centre (2024221–13434), Institute for Medical Research (IMR) and Sarawak, Malaysia (23–02751-FC8), Sri Lanka (EC-17–020), Cambodia (19IN109), Darwin, Australia (HREC 04/09), and Townsville, Australia (HREC/QTHS/87949). Written informed consent was obtained from all participants in Thailand, Lao PDR, Vietnam, Sarawak in Malaysia, Cambodia, and Darwin in Australia. Consent was not obtained from participants in Sri Lanka, Universiti Malaya Medical Centre and IMR in Malaysia, and Townsville in Australia, as all specimens were fully anonymized. These study protocols were additionally reviewed and approved by the Ethics Committee of Faculty of Tropical Medicine, Mahidol University (MUTM 2023-079-01).

### Development of a second-generation Hcp1-ICT

The second-generation Hcp1-ICT was developed using recombinant Hcp1 (rHcp1) [21] as the target antigen and was manufactured at AFFINOME Co., Ltd, Thailand. The optimal protein concentration for use in the assays was determined using pooled sera from ten culture-confirmed melioidosis patients and ten healthy Thai donors that was serially diluted (2-fold serial dilution, 1:2–1:8,192). rHcp1 was diluted with phosphate-buffered saline (PBS, pH 7.4) to 0.5, 0.8, 1.0, 1.2, and 1.5 mg/mL sprayed onto nitrocellulose membranes and dried. The conjugate pads were sprayed with colloidal gold-conjugated anti-human IgG and anti-chicken IgY. The control line was coated with chicken IgY. The membranes were cut and assembled into test strips along with a sample pad, a conjugate pad, and an absorbent pad in an environmentally controlled room. Serum or plasma samples were tested at various dilutions with the strips by adding 10 µL to the sample wells, followed by the addition of 3 drops (approximately 100 µL) of running buffer, and incubation at room temperature (RT) for 15 minutes. Following optimization of the various parameters, the test strips were prepared using the optimal protein concentrations and packaged in sealed aluminum pouches with silica gel desiccant for storage at 2–30 °C. Accelerated aging stability testing indicated an estimated real-time shelf life of approximately 24 months. The assay requires minimal equipment and simple visual interpretation, making it suitable for use in low-resource hospital settings. Prior to Hcp1-ICT testing, the strips were removed from the packaging, and the reactivity of individual serum samples was evaluated.

### Determination of the limit of detection of the second-generation Hcp1-ICT

To determine the limit of detection (LOD) of the Hcp1-ICT, positive serum samples from patients with culture-confirmed melioidosis were pooled and serially diluted (2-fold serial dilution, 1:2–1:16,384) using pooled negative sera from healthy individuals as a diluent, resulting in 15 different concentrations for testing. The diluted samples were then evaluated with the Hcp1-ICT in duplicate and results were compared to the results of an ELISA as described below. Only the LOD concentration (1:32 dilution) was repeated 20 times to confirm reproducibility.

### ELISA for anti-Hcp1 IgG detection

An indirect enzyme-linked immunosorbent assay (ELISA) was performed to quantify anti-Hcp1 IgG antibodies as previously described [25,28]. Briefly, 96-well plates were coated overnight at 4°C with recombinant Hcp1 protein (2.5 µg/mL) in carbonate-bicarbonate buffer (pH 9.6). Plates were washed and blocked with 5% skim milk in PBS for 2 hours at 37°C. Serum samples were diluted 1:2,000 in assay diluent and incubated for 30 minutes at room temperature. After washing, horseradish peroxidase-conjugated rabbit antihuman IgG (1:2,000 dilution) was added and incubated for 30 minutes. The reaction was developed using TMB substrate and stopped with 1N hydrochloric acid. Optical density was measured at 450 nm. All samples were tested in duplicate, and mean OD_450_ values were used for analysis.

### Clinical samples for initial evaluation of the second-generation Hcp1-ICT

**Fig 1:**
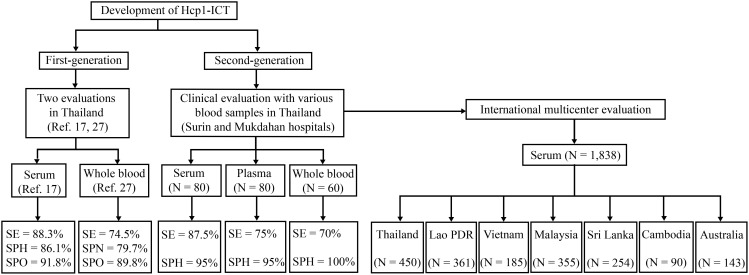
Flow diagram depicting the development and evaluation process for first- and second-generation Hcp1-ICT assay. Diagnostics performance was assessed in term of sensitivity (SE), specificity using samples with healthy (SPH), specificity using samples with other infections (SPO), specificity using samples from no pathogen-detected group (SPN). References (Ref. 17, Ref. 27) correspond to the studies cited in the reference section.

A total of 220 stored samples were used to evaluate the clinical performance of the second-generation Hcp1-ICT. These included 80 serum, 80 plasma, and 60 whole blood samples (Fig 1, Table 1). The serum and plasma samples each comprised 40 samples from culture-confirmed melioidosis patients and 40 from healthy donors. These samples were collected between 2021 and 2023 at Surin Hospital (N = 131) and Mukdahan Hospital (N = 89) in Thailand. The whole blood samples included 30 samples from culture-confirmed melioidosis patients and 30 from healthy subjects, collected at Mukdahan Hospital (N = 60) (Table 1). Serum samples were collected in clot activator tubes without anticoagulant, whereas plasma and whole blood specimens were collected in EDTA anticoagulant tubes. All serum, plasma, and whole blood samples were obtained from previous prospective studies and had been stored at –80 °C until testing in this study. These samples were used exclusively for the initial comparative evaluation and were not included in the international multicenter evaluation described below.

**Table 1:** Clinical samples and study sites included in the initial evaluation (N = 220) and the international evaluation (N = 1,838) of a second-generation Hcp-ICT.

Origin of samples	Type of sample	Number of samples			
		Culture-confirmed melioidosis	Other infections	Healthy donor	Total
**Initial evaluation**					
**Thailand**					
Mukdahan Hospital	Serum	6	ND	15	21
	Plasma	ND	ND	8	8
	Whole blood	30	ND	30	60
Surin Hospital	Serum	34	ND	25	59
	Plasma	40	ND	32	72
	Whole blood	ND	ND	ND	ND
Total		110	ND	110	220
**International evaluation**					
**Thailand**	Serum	150	150	150	450
Mukdahan Hospital		10	24	71	105
Roi-Et Hospital		37	7	60	104
Surin Hospital		103	119	19	241
**Lao PDR**	Serum	61	150	150^a^	361
Mahosot Hospital Vientiane					
**Vietnam**	Serum	63	62	60	185
VNU-Institute of Microbiology and Biotechnology					
**Malaysia**	Serum	142	134	79	355
Universiti Malaya Medical Centre (UM)		8	96	ND	104
Sarawak		5	38	79	122
Miri Hospital					
Bintulu Hospital					
Sarawak General Hospital					
Institute for Medical Research (IMR)		129	ND	ND	129
**Sri Lanka**	Serum	74	80	100	254
University of Colombo					
**Cambodia**	Serum	30	30	30	90
National Institutes of Health National Institute of Allergy and Infectious Diseases					
**Australia**	Serum	81	33	29	143
Royal Darwin Hospital		16	3	9	28
Townsville University Hospital		65	30	20	115
Total		601	639	598	1,838

^a^Serum samples were obtained from blood donors in Salavan (Saravane) province, located far from Vientiane, a region known for its high density of *B. pseudomallei* in soil and rivers [29]; ND, not done.

### Serum samples for international multicenter evaluation of the second-generation Hcp-ICT

Stored serum samples from 1,838 individuals across Thailand, Lao PDR, Vietnam, Malaysia, Sri Lanka, Cambodia and Australia were used to evaluate the performance of the second-generation Hcp1-ICT (Fig 1). The evaluation was conducted and included the following sets: (i) 601 sera from culture-confirmed melioidosis patients, (ii) 598 sera from healthy subjects, and (iii) 639 sera from patients with other infections (Table 1). The other organisms reported were isolated from the blood or other specimens from these patients by the hospitals as listed in S1 Table.

### Evaluation of the second-generation Hcp1-ICT using serum samples

**Fig 2:**
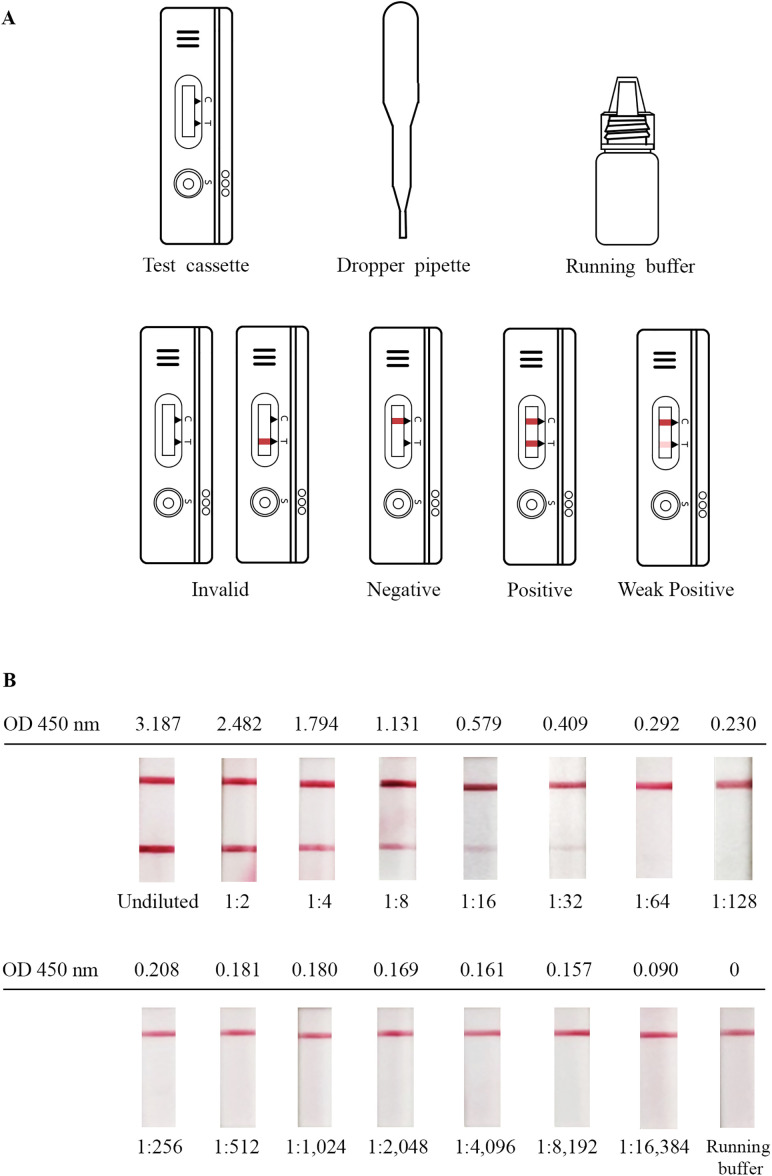
The second-generation of Hcp1-ICT. **(A)** Packaging contents and result interpretation of Hcp1-ICT. **(B)** Limit of detection of the Hcp1-ICT compared with Hcp1-ELISA [25,28], evaluated using serial dilutions of pooled melioidosis sera (1:2 to 1:16,384) and undiluted pooled healthy sera as diluent and negative control. Representative ELISA results are shown as optical density (OD) values measured at 450 nm (OD₄₅₀). Each concentration was tested in duplicate, and the 1:32 dilution was repeated 20 times to ensure reproducibility.

A 10 µL volume of serum was applied to the sample well (S) of the device followed by the addition of 3 drops of running buffer. Results were read by eye following incubation for 15 minutes at RT and interpreted as per Fig 2A. For positive results, two distinct red lines appeared, one in the control (C) region and one in the test (T) region, indicating the presence of anti-Hcp1 IgG antibodies. The intensity of the test line varied depending on the antibody concentration in the samples. A weak positive result showed a faint test line, indicating low levels of anti-Hcp1 IgG antibodies or possible non-specific cross-reactivity. For negative results, only the control (C) line appeared, indicating the absence of anti-Hcp1 IgG antibodies or levels below the detection threshold. If the control (C) line failed to appear, the result was considered invalid, and the procedure was reviewed before repeating the test with a new device. The performance of these assays was compared with bacterial culture results.

### Evaluation of the second-generation Hcp1-ICT for diagnosis of melioidosis

A total of 1,838 anonymous human serum samples were used to evaluate the second-generation Hcp1-ICT for diagnosing melioidosis (Fig 1). The device was evaluated at study sites in Thailand, Lao PDR, Vietnam, Malaysia, Sri Lanka, Cambodia, and Australia. The sets of serum samples used for evaluation of Hcp1-ICT are listed in Table 1 and S1 Table.

### Statistical analysis

Data were analyzed using IBM SPSS Statistics for Windows, version 29.0 (IBM Corp., Armonk, NY, USA), and GraphPad Prism, version 10.3.1 (GraphPad Software, San Diego, CA, USA). Kappa and McNemar tests were used to assess the agreement between different detection methods. Differences were considered significant when the *P*-value was < 0.05. The accuracy, sensitivity, specificity, positive predictive value (PPV), and negative predictive value (NPV), positive likelihood ratio (+LR), negative likelihood ratio (−LR), and accuracy of the second-generation Hcp1-ICT was calculated using culture as the reference method. Ninety-five percent confidence intervals (CIs) were calculated using the exact Clopper-Pearson method.

Sensitivity = a/(a + c)
Specificity = d/(b + d)
Positive predictive value (PPV) = a/(a + b)
Negative predictive value (NPV) = d/(c + d)
Positive likelihood ratio (+LR) = [a/ (a + c)]/[1 − d/(b + d)]
Negative likelihood ratio (−LR) = [1 − a/(a + c)]/[d/(b + d)]
Accuracy = (a + d)/(a+ b + c + d)

A kappa test was also used to determine concordance between Hcp1-ICT results and culture results. The kappa value was determined using the formula:

Kappa value (κ) = (P_o_ - P_e_)/ (1 - P_e_)
P_o_ = (a + d)/(a + b + c + d)
P_e_ = [(a + c)(a + b)+(b + d)(c + d)]/(a + b + c + d)^2^

where a = true positives, b = false positives, c = false negatives and d = true negatives.

## Results

### Development and evaluation of a second-generation Hcp1-ICT

Fig 1 shows the flow diagram outlining the development and evaluation process of the first- and second-generation Hcp1-ICT assays. The first-generation Hcp1-ICT, previously evaluated in Thailand, showed a sensitivity of 88.3% and specificities of 86.1% and 91.8% with serum samples from healthy donors and patients with other infections [17]. However, its performance with whole blood was lower (74.5%) with specificities of 79.7% and 89.8% in samples from no pathogen-detected group and with other infections, respectively [27] (Fig 1).

In the present study, a second-generation Hcp1-ICT was developed and manufactured on a large scale by a company with ISO 13485 standards, and used optimized reagents and an improved assay design to enhance diagnostic performance. The kit consisted of the test devices, dropper pipettes, running buffer and an instruction manual (Fig 2A). The limit of detection (LOD) was defined as the lowest dilution of pooled positive serum that consistently yielded positive results. Using this criterion, the LOD was determined to be a 1:32 dilution. All 20 independent repeat tests showed the same result, with positive results observed at this dilution, confirming the reproducibility of the assay (Fig 2B). Pooled serum was prepared from archived serum samples obtained from culture-confirmed melioidosis patients. Individual samples were previously tested for anti-Hcp1 IgG by ELISA as previously described [25,28], and only ELISA-positive samples (OD_450_ ≥ 1.165) were included. Equal volumes of these ELISA-positive serum samples were combined to generate the pooled serum used for serial dilution and LOD determination. ELISA was performed in duplicate, and background-subtracted OD_450_ values were calculated. The ELISA positivity cutoff was defined as a background-subtracted OD_450_ value of 0.3, based on its clear separation from negative control values. This cutoff was assigned a value of 1 arbitrary Unit/mL, and antibody levels were calculated by dividing background-subtracted OD_450_ values by 0.3. Using this conversion, the dilution corresponding to an OD_450_ of 0.41 was equivalent to 1.36 Unit/mL, indicating the LOD for the Hcp1-ICT was determined to be 1.36 Units/mL (Fig 2B). The ELISA results also showed a consistent decrease in OD_450_ values with increasing serum dilution, supporting the reliability of the assay and its use as a reference method for defining the detection threshold of the Hcp1-ICT (S2 Table).

### Clinical evaluation of the second-generation of Hcp1-ICT across blood samples types

A total of 220 blood samples were collected from 220 patients with culture-confirmed melioidosis, culture-confirmed other infections, and healthy donors, as part of a previous prospective study conducted at Surin and Mukdahan Hospitals in Thailand [30, Brett, PJ et al, manuscript in preparation]. The samples comprised serum (N = 80), plasma (N = 80), and whole blood (N = 60) (Fig 1, Table 1). This evaluation assessed assay performance in real-world clinical settings, ensuring reliable detection of melioidosis across multiple sample types and clinical conditions (Table 2).

**Table 2:** Clinical performance of second-generation Hcp1-ICT using serum, plasma, and whole blood samples from Thailand, with culture as the reference standard. The analysis included 110 patients with melioidosis and 110 healthy subjects.

Type of sample	% Sensitivity (95% Cl)	% Specificity in healthy samples (95% Cl)	% PPV (95% Cl)	% NPV (95% Cl)	% Accuracy (95% Cl)
Serum	87.5 (35/40) (73.2 – 95.8)	95 (38/40) (83.1 – 99.4)	94.6 (35/37) (81.9 – 95.6)	88.4 (38/43) (76.9 – 94.5)	91.3 (73/80) (82.8 – 96.4)
Plasma	75 (30/40) (58.8 – 87.3)	95 (38/40) (83.1– 99.4)	93.8 (30/32) (79.3 – 98.3)	79.2 (38/48) (68.9 – 86.7)	85 (68/80) (75.3 – 92)
Whole blood	70 (21/30) (50.6 – 85.3)	100 (30/30) (88.4 – 100)	100 (21/21) (83.9 – 100)	76.9 (30/39) (65.9 – 85.2)	85 (51/60) (73.4 – 92.9)

PPV, positive predictive value; NPV, negative predictive value; CI, confidence interval.

Serum samples demonstrated the highest diagnostic performance with a sensitivity of 87.5% and specificity of 95.0% among healthy individuals. The positive predictive value (PPV) and negative predictive value (NPV) were 94.6% and 88.4%, respectively, yielding an overall diagnostic accuracy of 91.3%.

Plasma samples exhibited moderate sensitivity at 75.0% with specificities of 95.0% for healthy individuals. The PPV was 93.8%, the NPV was 79.2% and the accuracy for plasma testing was 85.0%.

Whole blood samples had the lowest sensitivity at 70.0%, but achieved 100% specificity in healthy controls. The PPV was 100%, the was NPV of 76.9%, and the overall accuracy was 85.0%.

These findings indicate that serum samples performed best, whereas plasma and whole blood had high specificity but reduced sensitivity.

### International evaluation of rapid second-generation Hcp1-ICT for diagnosis of melioidosis

**Fig 3:**
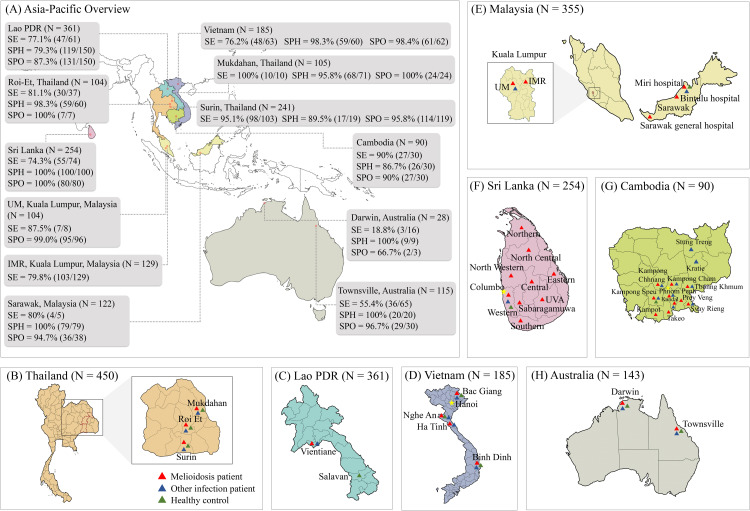
(A) Overview of the Asia–Pacific region showing participating countries. The sensitivity (SE), specificity using samples from healthy controls (SPH), and specificity using samples from patients with other infections (SPO) of the second-generation Hcp1-ICT evaluated at study sites in Thailand, Lao PDR, Vietnam, Malaysia, Sri Lanka, Cambodia, and Australia are shown. UM, Universiti Malaya Medical Centre; IMR, Institute for Medical Research. **(B)** Thailand: samples from melioidosis patients, patients with other infections, and healthy controls were collected at Mukdahan, Surin, and Roi-Et hospitals. **(C)** Lao PDR: samples from melioidosis patients and patients with other infections were collected in Vientiane, while healthy controls were collected in Salavan (Saravane). **(D)** Vietnam: samples from melioidosis patients and patients with other infections were collected in Bac Giang, Nghe An, Ha Tinh, and Binh Dinh provinces, while healthy controls were collected in Bac Giang, Nghe An, and Binh Dinh. **(E)** Malaysia: samples from melioidosis patients were collected at Universiti Malaya Medical Centre (UM), Institute for Medical Research (IMR), and Miri, Bintulu, and Sarawak General hospitals. Samples from patients with other infections were collected at UM and Miri hospital, while healthy controls were collected at Miri hospital. **(F)** Sri Lanka: samples from melioidosis patients were collected across multiple provinces including Northern, North Central, North Western, Eastern, Central, UVA, Sabaragamuwa, Western, and Southern provinces. Samples from patients with other infections and healthy controls were collected in the Western province. **(G)** Cambodia: samples from melioidosis patients were collected across several provinces including Kampong Chhnang, Kampong Cham, Tboung Khmum, Kampong Speu, Phnom Penh, Kandal, Prey Veng, Svay Rieng, Kampot, and Takeo. Samples from patients with other infections were collected in Stung Treng, Kratie, Kampong Chhnang, Kampong Cham, Tboung Khmum, Kampong Speu, Phnom Penh, Kandal, and Svay Rieng, while healthy controls were collected in Kampong Speu. **(H)** Australia: samples from patients with melioidosis, patients with other infections, and healthy controls were collected in Darwin and Townsville.

To further establish the robustness and utility of the second-generation Hcp1-ICT, an international multicenter evaluation was conducted using 1,838 serum samples obtained from patients and healthy donors in Thailand, Lao PDR, Vietnam, Malaysia, Sri Lanka, Cambodia, and Australia. This large-scale evaluation aimed to determine the performance of the assay across different geographic regions, accounting for potential variations in strain prevalence, host immune responses and clinical settings (Figs 1, 3, Table 3). The geographic distribution of the samples, including the regions or provinces from which the participants were recruited, is shown in Fig 3B-3H.

**Table 3:** Diagnostic performance of the second-generation Hcp1-ICT, including sensitivity, specificity, positive predictive value, negative predictive value, accuracy, and corresponding 95% confidence intervals across Thailand, Lao PDR, Vietnam, Malaysia, Sri Lanka, Cambodia and Australia.

Country	% Sensitivity (95% Cl)	% Specificity in healthy samples (95% Cl)	% Specificity in samples with other infections (95% Cl)	% PPV (95% Cl)	% NPV (95% Cl)	+LR (95% Cl)	-LR (95% Cl)	% Accuracy (95% Cl)	Kappa (95% Cl)
Thailand	92 (138/150) (86.4 – 95.8)	96 (144/150) (91.5 – 98.5)	96.7 (145/150) (92.4 – 98.9)	95.8 (138/144) (91.3 – 98.1)	92.3 (144/156) (87.4 – 95.4)	23 (0.92/0.04) (10.5 – 50.5)	0.1 (0.08/0.96) (0.05 – 0.14)	94 (282/300) (90.7 – 96.4)	0.88 (0.44/0.50) (0.83–0.93)
Lao PDR	77.1 (47/61) (64.5 – 86.9)	79.3 (119/150) (72 – 85.5)	87.3 (131/150) (80.9 – 92.2)	60.3 (47/78) (51.9 – 68.1)	89.5 (119/133) (84.2 – 93.1)	3.7 (0.77/0.21) (2.7 – 5.2)	0.3 (0.23/0.79) (0.2 – 0.5)	78.7 (166/211) (72.5 – 84)	0.52 (0.23/0.45) (0.4 – 0.64)
Vietnam	76.2 (48/63) (63.8 – 86)	98.3 (59/60) (91.1 – 100)	98.4 (61/62) (91.3 – 100)	98 (48/49) (87.2 – 99.7)	79.7 (59/74) (71.6 – 86)	45.7 (0.76/0.02) (6.5 – 320.8)	0.2 (0.24/0.98) (0.2 – 0.4)	87 (107/123) (79.7 – 92.4)	0.74 (0.37/0.50) (0.63 – 0.86)
Malaysia	80.3 (114/142) (72.8 – 86.5)	100 (79/79) (95.4 – 100)	97.8 (131/134) (93.6 – 99.5)	100 (114/114) (96.8 – 100)	73.8 (79/107) (66.9 – 79.7)	∞	0.2 (0.2/1) (0.1 – 0.3)	87.3 (193/221) (82.2 – 91.4)	0.74 (0.37/0.50) (0.66 – 0.83)
Sri Lanka	74.3 (55/74) (62.8 – 83.8)	100 (100/100) (96.4 – 100)	100 (80/80) (95.5 – 100)	100 (55/55) (93.5 – 100)	84 (100/119) (78.1 – 88.6)	∞	0.3 (0.26/1) (0.2 – 0.4)	89.1 (155/174) (83.5 – 93.3)	0.77 (0.36/0.47) (0.67 – 0.86)
Cambodia	90 (27/30) (73.5 – 97.9)	86.7 (26/30) (69.3 – 96.2)	90 (27/30) (73.5 – 97.9)	87.1 (27/31) (72.9 – 94.4)	89.7 (26/29) (74.6 – 96.2)	6.8 (0.90/0.13) (2.69 – 16.94)	0.1 (0.1/0.87) (0.04 – 0.3)	88.3 (53/60) (77.4 – 95.2)	0.77 (0.38/0.50) (0.6 – 0.93)
Australia	48.1 (39/81) (36.9 – 59.5)	100 (29/29) (88.1 – 100)	93.9 (31/33) (79.8 – 99.3)	100 (39/39) (91.0 – 100)	40.9 (29/71) (35.9 – 46.0)	∞	0.5 (0.5/1) (0.4 – 0.6)	61.8 (68/110) (52.1 – 70.9)	0.33 (0.19/0.57) (0.21 – 0.45)
Total	77.9 (468/601) (74.3 – 81.1)	93.0 (556/598) (90.6 – 94.9)	94.8 (606/639) (92.8 – 96.4)	91.8 (468/510) (89.3 – 93.7)	80.7 (556/689) (78.2 – 83.0)	11.1 (0.78/0.07) (8.26 – 14.9)	0.24 (0.22/0.93) (0.2 – 0.3)	85.4 (1024/1199) (83.3 – 87.4)	0.71 (0.35/0.50) (0.67 – 0.75)

PPV, positive predictive value; NPV, negative predictive value; + LR, positive likelihood ratio; -LR, negative likelihood ratio; CI, confidence interval. PPV, NPV, LRs, accuracy and Kappa were calculated using data from patients with melioidosis and healthy samples. ∞ , perfect specificity (no false positives).

**Thailand** demonstrated the highest overall diagnostic performance with a sensitivity of 92.0%, specificity of 96.0% using serum samples from healthy individuals, and 96.7% among patients with other infections. The PPV and NPV reached 95.8% and 92.3%, respectively, with an overall accuracy of 94% and a kappa coefficient of 0.88, indicating strong agreement with from culture results. Across different study sites within Thailand, the second-generation Hcp1-ICT showed some variation in performance. The sensitivity of the assay was 100% using samples from Mukdahan, 81.1% using samples from Roi-Et, and 95.1% using samples from Surin. The specificity with samples from healthy individuals from the same regions was 95.8%, 98.3%, and 89.5%, respectively. Similarly, the specificity using samples from patients with other infections was 100%, 100%, and 95.8% (Fig 3).

**Lao PDR** showed sensitivity at 77.1% with specificities of 79.3% using samples from healthy controls (from Salavan province, located far from Vientiane) and 87.3% using samples from patients with other infections. The PPV and NPV were 60.3% and 89.5%, respectively, yielding an accuracy of 78.7% and moderate agreement with culture results (κ = 0.52).

**Vietnam** achieved a sensitivity of 76.2% with notably high specificity of 98.3% and 98.4% using healthy and other infection samples, respectively. The PPV was 98.0% and the NPV 79.7%, resulting in an accuracy of 87.0% and a kappa value of 0.74.

**Malaysia** exhibited a sensitivity of 80.3%, with specificities of 100% using healthy samples and 97.8% among samples from patients with other infections. Both the PPV and NPV were high (PPV: 100% and NPV: 73.8%), giving an accuracy of 87.3% and kappa of 0.74. Across different Malaysian study sites, the sensitivity was highest at Universiti Malaya Medical Centre (UM) (87.5%), followed by Institute for Medical Research (IMR) (79.8%) and Sarawak (80.0%). The specificity in healthy individuals was 100% at Sarawak but was not assessed at UM or IMR. The specificity in patients with other infections was 99.0% at UM and 94.7% at Sarawak (Fig 3).

**Sri Lanka** reported a sensitivity of 74.3% with 100% specificity using samples from both healthy donors and patients with other infections. All 80 samples from patients with infections other than melioidosis tested negative, confirming the high diagnostic specificity of the assay (100%). This resulted in PPV and NPV values of 100% and 84.0%, respectively, with an accuracy of 89.1% and a kappa coefficient of 0.77.

**Cambodia** achieved high sensitivity at 90.0% with specificities of 86.7% and 90.0% using healthy donor and other infection samples, respectively. The PPV and NPV were 87.1% and 89.7%, respectively, resulting in an overall accuracy of 88.3% and a kappa value of 0.77.

**Australia** displayed the lowest level of sensitivity at 48.1% despite 100% specificity in healthy individuals and 93.9% specificity among patients with other infections. While the PPV reached 100%, the low NPV of 40.9% led to an overall accuracy of 61.8% and a kappa value of 0.33. Across different study sites, the sensitivity was higher in Townsville (55.4%) than in Darwin (18.8%). The specificity in healthy individuals was 100% at both sites, whereas specificity in patients with other infections was 96.7% for Townsville and 66.7% for Darwin (Fig 3).

Likelihood ratios (LRs) indicate how much more likely a positive test result is in patients with the disease than in those without it. LRs inform clinicians of a patient’s probability of having melioidosis based on the pre-test probability. In the present study, + LR across seven countries ranged from 3.7 to 45.7, indicating a 3.7 to 45.7-fold increase in the odds of melioidosis-infected patient testing positive with the second-generation Hcp1-ICT. In Malaysia, Sri Lanka, and Australia, the + LR was infinite (∞), indicating perfect specificity (no false positives) and indicating that a positive test result confirms the presence of the disease. A value of +LR that is greater than one and –LR that is less than one obtained by the second-generation Hcp1-ICT compared to the culture indicates that the second-generation Hcp1-ICT developed in this study has the potential to be a useful diagnostic tool for melioidosis.

Taken together, these results show that the second-generation Hcp1-ICT demonstrated excellent performance, with +LR > 1 and -LR < 1 across all six countries, and sensitivity consistently 74–92%. In contrast, testing in Australia, showed reduced assay performance (sensitivity 48.1%) despite very high specificity (Table 3, Fig 3).

Across all serum samples, the second-generation Hcp1-ICT showed a sensitivity of 77.9% and a specificity of 93.0% in healthy individuals, with 94.8% specificity among patients with other infections. The PPV and NPV were 91.8% and 80.7%, respectively. Diagnostic accuracy was 85.4%, with substantial agreement with culture results (κ = 0.71) (Table 3).

To assess the specificity of the second-generation Hcp1-ICT, serum samples from patients with non-melioidosis infections as identified by culture, serology and molecular methods across all study sites were tested. Of the 639 samples, only 12 (1.9%) were positive and 21 (3.3%) were weakly positive (S1 Table). Positive and weakly positive results were distributed across a range of bacterial and viral pathogens, including *Acinetobacter baumannii*, *Enterobacter cloacae, Escherichia coli*, *Klebsiella pneumoniae*, *Proteus mirabilis, Pseudomonas* sp*., Staphylococcus* spp., *Streptococcus constellatus* and arboviruses such as chikungunya and dengue viruses. The highest numbers of weakly positive results were detected in infections with *E. coli* (N = 7), *S. aureus* (N = 5), and *K. pneumoniae* (N = 2); however, BLASTP analysis (https://blast.ncbi.nlm.nih.gov/Blast.cgi) confirmed no sequence homology between rHcp1 and the genomes of these organisms, indicating a minimal risk of cross-reactivity.

## Discussion

Bacterial culture is the gold standard for melioidosis diagnosis but is limited by reduced sensitivity and slow turnaround [31–33]. The IHA also has well-known limitations, including high background seropositivity, poor sensitivity in early infection, and lack of specificity for active disease [18,34–38]. These constraints highlight the need for rapid POC diagnostics. In clinical practice, diagnostic strategies are guided by the patient’s presentation, and the combined use of rapid serological testing together with culture may improve diagnostic confidence. This study aimed to improves upon our Hcp1-ICT for the diagnosis of melioidosis. In a comparative study in Thailand (Surin and Mukdahan)**,** the second-generation Hcp1-ICT showed high accuracy with serum (sensitivity 87.5%, specificity 95%). With serum samples, sensitivities ranged from 74% to 92% across six countries, with specificities consistently being 79–100% in healthy controls, with substantial agreement with culture (κ = 0.52–0.88), except in Australia, where agreement was lower (κ = 0.33; sensitivity 48%).

Our initial evaluation showed that the second-generation Hcp1-ICT achieved a LOD of 1.36 arbitrary units/mL of anti-Hcp1 IgG in serum. In serum samples, it improved specificity to 95% compared with the first-generation assay while maintaining high sensitivity. Although plasma was not previously assessed, specificity was consistent in healthy donors. In whole blood, the first-generation assay had low sensitivity, whereas the second-generation improved specificity from 79.7% to 100%, highlighting a clear performance advantage. This improvement likely reflects assay optimization (flow rate, blocking, buffer formulation, and gold conjugation), which reduced background and enhanced signal-to-noise ratio. Standardized manufacturing further ensured batch consistency and stability, supporting reliable POC use.

The second-generation assay demonstrated high sensitivity with serum, but reduced sensitivity in plasma (87.5% vs. 75%) and whole blood (87.5% vs. 70%) relative to culture as the gold standard. These differences are partly attributable to sample type. Inappropriate specimen processing or storage can alter sample properties over time and affect results [39]. In addition, fibrinogen and coagulation factors in plasma and whole blood can interfere with antigen-antibody binding and increase background signals in immunoassays [39, 40]. Serum, free of coagulation factors, provides greater stability and fewer matrix effects, supporting routine in vitro diagnostics [41]. In contrast, fibrinogen can hinder antigen–antibody interactions, reducing sensitivity and reproducibility; similar effects have been reported in other assays, including cortisol, digoxin, alpha-fetoprotein, and prostate-specific antigen [39,42,43]. Collectively, these findings support serum as the optimal sample type for the second-generation Hcp1-ICT, providing the highest sensitivity and most consistent diagnostic performance and justifying its selection for international evaluation.

An international multicenter evaluation confirmed that the second-generation Hcp1-ICT provides robust diagnostic accuracy for melioidosis, with high sensitivity together with strong specificity in Southeast Asia, while performance was notably lower in Australia. Among the study sites, Thailand, Vietnam, Malaysia, Sri Lanka, and Cambodia demonstrated the highest concordance with culture (κ = 0.74–0.88), highlighting the reliability of the assay in endemic areas. Overall, the assay showed high specificity (93.0%) and substantial agreement with the reference standard (κ = 0.71), with a strong positive likelihood ratio (+LR = 11.1), supporting its utility as a POC diagnostic tool. Sensitivity was moderate (77.9%) and varied between countries, likely reflecting differences in disease stage, host response, and background seroepidemiology [16,34,44], while consistently high specificity across sites indicates a low risk of false-positive results. Further optimization could include improved antigen orientation, incorporation of additional immunodominant *B. pseudomallei* antigens, refinement of anti-human IgG–gold nanoparticle ratios, increased signal intensity, and adoption of double-antigen bridging formats to enhance antibody capture while minimizing nonspecific binding.

Site-specific performance was highest in Mukdahan, with Roi-Et and Surin also performing well, supporting the overall high diagnostic accuracy in Thailand, consistent with previous reports [17]. The high sensitivity in Thailand may reflect relatively later sampling during the course of illness, suggesting that patients presented at a stage of illness when anti-Hcp1 IgG responses were likely already detectable. In Malaysia, comparable results were observed across UM, IMR, and Sarawak. Specificity was lowest in Lao PDR, where samples from Salavan province, an area with high environmental *B. pseudomallei* density [29], suggested increased false positivity. Sensitivity in Australia was lower, with Townsville outperforming Darwin, likely due to earlier sampling in Darwin during the acute phase, when antibody responses were still developing, making anti-Hcp1 IgG difficult to detect, especially in those with only a few days of symptom prior to admission.

Serology is often negative early in the illness for melioidosis as it is for most infections [18,45–47]. This is supported by low or undetectable IHAT titers (<1:20) in most Darwin patients, indicating limited antibody responses at sampling (S3 Table), which may lead to false-negative results, whereas later sampling in Townsville corresponded with higher titers and improved detection.

Differences in disease presentation may also contribute, as higher rates of bacteraemia in Thailand (68.7%; 103/150) compared with Darwin (56.3%; 9/16) (S4 Table) may reflect greater systemic bacterial burden and stronger antibody responses, enhancing assay sensitivity. In contrast, patients with non-bacteraemia may have lower circulating antibody levels, potentially reducing sensitivity. The high proportion of bacteraemia in Townsville (100%; 65/65) likely reflects the characteristics of the archived sample set rather than the true distribution of clinical presentations. Regional seroepidemiological differences may also influence assay performance. In Southeast Asia, frequent environmental exposure to *B. pseudomallei*, particularly during agricultural activities in the rainy season, results in higher baseline antibody levels, whereas lower background seropositivity is observed in northern Australia [16,18,48–50]. Variations in exposure, potential cross-reactivity with environmental *Burkholderia* species, and strain virulence [16,50] may contribute to differences in assay sensitivity. Although accurate timing of serum sampling relative to illness onset was unavailable, prospective studies with timed serial sampling are needed to determine whether lower sensitivity in Australia reflects sampling timing or regional Hcp1 variation [23]. Regional variability may also reflect sampling when anti-Hcp1 IgG levels are below the LOD, as well as host factors such as patient immune response, particularly in immunocompromised patients [17], stage of illness, and sample handling, including prolonged storage and repeated freeze–thaw cycles that reduce IgG stability [17,51–53]. In addition, storage outside the recommended temperature range (2–30 °C) may compromise assay performance by affecting gold conjugates and antibody binding on nitrocellulose membranes [54]. Despite lower sensitivity in some settings, the second-generation Hcp1-ICT maintained high specificity in healthy controls, supporting the validity of the test results.

Our findings are consistent with previous reports showing that diagnostic performance varies across regions due to differences in sample quality, healthcare practices, and population characteristics, with spectrum bias further influencing sensitivity and specificity [55]. The first-generation Hcp1-ICT has been shown to detect culture-negative cases, with anti-Hcp1 IgG detectable from 3 to 155 days after symptom onset [26], and higher detection rates in patients with ≥7 days of symptoms compared with 1–6 days, supporting its role in early anti-Hcp1 IgG detection [27]. Among culture-negative patients with strong clinical suspicion of melioidosis, the Hcp1-ICT identified anti-Hcp1 IgG antibodies in a significant proportion of cases, supporting its value as a complementary tool when culture fails [27]. These findings highlight its clinical utility for early detection and case management where culture sensitivity is limited.

Recently, Tandhavanant et al. [23] identified nine Hcp1 variants among 1,283 clinical isolates, with three types detected in northeast Thailand. The predominant type, identical to K96243, accounted for 98.1% of isolates, while variant A (1.9%) was also reported in Laos, Vietnam, India, and Australia, likely linked to environmental reservoirs. A rare variant B, newly identified in Thailand, has also been found in Australia and the USA, indicating sporadic global distribution. Different Hcp1 variants influence melioidosis patients’ antibody responses and the ability to induce multinucleated giant cell formation, both Hcp1 variation and local strain distribution should be considered when interpreting Hcp1-based diagnostic performance across regions. In this study, comparative sequencing of infecting strains was not performed because only archived sera without paired bacterial isolates were available. Therefore, the relationship between Hcp1 sequence variation and regional differences in assay sensitivity could not be assessed. While geographic variation in Hcp1 remains a possible explanation, other factors including host immune variability, timing of sample collection, disease severity, and specimen storage conditions may also have contributed [56–58]. Prospective studies with paired isolate sequencing and detailed clinical data are needed to clarify this.

Among patients with non-melioidosis infections, low rates of positive and weakly positive results (1.9% and 3.3%) were observed, with BLASTP confirming no homology between rHcp1 and these non-*B. pseudomallei* organisms. Specimen collection time points was unavailable, and prolonged hospitalization with antibiotics may have contributed to negative *B. pseudomallei* cultures in these patients [17]. Some seropositivity may have resulted from previous *B. pseudomallei* infections from which patients had recovered, or from exposure without active infection. Despite this, the assay demonstrated excellent specificity in healthy donors, including those from highly endemic areas as counter-evidence. Although culture remains the gold standard, it is limited by slow turnaround, infrastructure requirements, and reduced sensitivity after antibiotics or with suboptimal specimens [4,16,32], and IHA shows low early sensitivity and high background seropositivity [18,48,59]. The second-generation Hcp1-ICT offers rapid turnaround, ease of use, and minimal laboratory requirements, and while it cannot replace culture, it serves as a valuable adjunct for early diagnosis and clinical management when interpreted alongside clinical and microbiological findings, particularly in resource-limited settings or when culture is delayed.

A key limitation of antibody-based tests, including the second-generation Hcp1-ICT, is that they detect host immune responses rather than the organism itself. Consequently, the assay cannot reliably distinguish active infection from prior exposure or resolved disease, particularly in endemic regions with common background seropositivity. Residual antibodies may persist after recovery, while early or immunocompromised patients may show delayed or absent responses, potentially causing false negatives. Therefore, results should be interpreted alongside clinical and microbiological findings.

This study also relied on stored serum samples, which may have affected antibody stability and assay sensitivity due to variable storage duration, freeze–thaw cycles, or sample integrity. Samples were collected at different time points and not specifically for this evaluation, potentially influencing anti-Hcp1 IgG levels and detection rates. Archived samples may not fully reflect real-time performance, and differences in sample size, disease prevalence, and patient characteristics across countries further limit generalizability. Prospective studies with well-documented collection times, paired fresh samples, and clinical data, together with comparisons of fresh and stored samples under varying conditions, are needed to better assess assay performance.

Our findings indicate that the second-generation Hcp1-ICT offers promising diagnostic performance for melioidosis, with improved specificity over earlier versions. While culture remains the reference standard, the Hcp1-ICT is rapid, user-friendly, and suitable for serological screening in resource-limited settings without specialized equipment or training. The second-generation Hcp1-ICT has been approved by the Thai FDA and manufactured under ISO 13485 standards, it supports reliable clinical and field use in endemic regions. Prospective studies across various geographic settings are warranted to further evaluate its diagnostic utility and support wider implementation in both referral centers and resource-limited environments.

## Supporting information

S1 Table Serum samples of non-*B. pseudomallei* organisms confirmed by culture, rapid diagnostic kit, or PCR assay were included in the international evaluation of second-generation across Thailand, Lao PDR, Vietnam, Malaysia, Sri Lanka, Cambodia, and Australia (N = 639). (DOCX)

S2 Table Determination of the limit of detection (LOD) of the second-generation Hcp1-ICT using serial dilutions of pooled melioidosis patient serum and comparison with ELISA measurements. (DOCX)

S3 Table Clinical and serological characteristics of culture-confirmed melioidosis patients from the Australian cohorts included in the evaluation of the second-generation of Hcp1-ICT. (DOCX)

S4 Table Specimen sources of *B. pseudomallei* among culture-confirmed melioidosis patients included in the study. (DOCX)

S1 Data Raw dataset used for the international multicenter evaluation of the second-generation Hcp1-ICT. (XLSX)
