# Supplementary material for: Development and international multicenter evaluation of a second-generation immunochromatography test for the serological diagnosis of melioidosis
Source: PLoS Negl Trop Dis. 2026 Jul 6;20(7):e0014484. doi: 10.1371/journal.pntd.0014484 (PMC13379097; doi:10.1371/journal.pntd.0014484)
Supplement: S1 Table — (DOCX) [file pntd.0014484.s001.docx]

**S1 Table** Serum samples of non-*B. pseudomallei* organisms confirmed by culture, rapid diagnostic kit, or PCR assay were included in the international evaluation of second-generation across Thailand, Lao PDR, Vietnam, Malaysia, Sri Lanka, Cambodia, and Australia (N = 639).

| **Strain** | **Total no. of serum samples** | **No. (%) of Hcp1-ICT-positive samples** | **No. (%) of Hcp1-ICT-negative samples** |
| --- | --- | --- | --- |
| *Achromobacter xylosoxidans* | 1 | 0 | 1 |
| *Acinetobacter baumannii* | 15 | 1 | 14 |
| *Acinetobacter* sp. | 4 | 0 | 4 |
| β-hemolytic streptococci | 1 | 0 | 1 |
| *Bacteroides fragilis* | 1 | 0 | 1 |
| *Bacteroides* sp. | 1 | 0 | 1 |
| *Bacteroides uniformis* | 1 | 0 | 1 |
| *Burkholderia cepacia* | 4 | 0 | 4 |
| *Candida orthopsilosis* | 1 | 0 | 1 |
| Chikungunya virus | 10 | 2 | 8 |
| *Citrobacter koseri* | 1 | 0 | 1 |
| Dengue virus | 102 | 2 | 100 |
| *Elizabethkingia anophelis* | 1 | 0 | 1 |
| *Enterococcus gallinarum* | 1 | 0 | 1 |
| *Enterobacter cloacae* | 3 | 1 | 2 |
| *Enterococcus casseliflavus* | 1 | 0 | 1 |
| *Enterococcus faecalis* | 7 | 0 | 7 |
| *Escherichia coli* | 219 | 10 | 209 |
| *Haemophilus influenzae* | 2 | 0 | 2 |
| *Klebsiella aerogenes* | 2 | 0 | 2 |
| *Klebsiella interoganes* | 2 | 0 | 2 |
| *Klebsiella oxytoca* | 1 | 0 | 1 |
| *Klebsiella pneumoniae* | 83 | 4 | 79 |
| *Klebsiella* sp. | 1 | 0 | 1 |
| *Leptospira interrogans* | 2 | 0 | 2 |
| *Morganella morganii* | 1 | 0 | 1 |
| *Mycobacterium abscessus* subsp. *massiliense* | 1 | 0 | 1 |
| *Mycobacterium tuberculosis* | 14 | 0 | 14 |
| *Pandorea anapnoica* | 1 | 0 | 1 |
| *Pasteurella multocida* | 1 | 0 | 1 |
| *Plasmodium vivax* | 3 | 0 | 3 |
| *Proteus mirabilis* | 6 | 1 | 5 |
| *Pseudomonas aeruginosa* | 19 | 0 | 19 |
| *Pseudomonas putida* | 1 | 0 | 1 |
| *Pseudomonas* sp. | 2 | 1 | 1 |
| *Salmonella enterica* | 2 | 0 | 2 |
| *Salmonella* sp. | 5 | 0 | 5 |
| *Salmonella* Typhi | 2 | 0 | 2 |
| *Serratia marcescens* | 1 | 0 | 1 |
| *Sphingobacterium multivorum* | 1 | 0 | 1 |
| *Staphylococcus aureus* | 87 | 8 | 79 |
| *Staphylococcus epidermidis* | 5 | 0 | 5 |
| *Staphylococcus haemolyticus* | 3 | 1 | 2 |
| *Staphylococcus hominis* | 6 | 1 | 5 |
| *Streptococcus agalactiae* | 4 | 0 | 4 |
| *Streptococcus constellatus* | 1 | 1 | 0 |
| *Streptococcus dysgalactiae* subsp. *equisimilis* | 1 | 0 | 1 |
| *Streptococcus pneumoniae* | 2 | 0 | 2 |
| *Streptococcus pyogenes* | 1 | 0 | 1 |
| *Streptococcus suis* | 1 | 0 | 1 |
| Viridans streptococci | 1 | 0 | 1 |
| Total | 639 | 33 (5.2) | 606 (94.8) |
