# Supplementary material for: Development and international multicenter evaluation of a second-generation immunochromatography test for the serological diagnosis of melioidosis
Source: PLoS Negl Trop Dis. 2026 Jul 6;20(7):e0014484. doi: 10.1371/journal.pntd.0014484 (PMC13379097; doi:10.1371/journal.pntd.0014484)
Supplement: S2 Table — (DOCX) [file pntd.0014484.s002.docx]

**S2 Table** Determination of the limit of detection (LOD) of the second-generation Hcp1-ICT using serial dilutions of pooled melioidosis patient serum and comparison with ELISA measurements.

| **Serum dilution** | **Test line appearance** | **Interpretation** | **OD_450_** | **ELISA Units (EU/mL)** |
| --- | --- | --- | --- | --- |
| Undiluted |  | Positive | 3.187 | 10.624 |
| 1:2 |  | Positive | 2.482 | 8.273 |
| 1:4 |  | Positive | 1. 794 | 5.978 |
| 1:8 |  | Positive | 1.131 | 3.768 |
| 1:16 |  | Positive | 0.579 | 1.930 |
| 1:32 |  | Positive | 0.409 | 1.363 |
| 1:64 |  | Negative | 0.292 | 0.974 |
| 1:128 |  | Negative | 0.230 | 0.767 |
| 1:256 |  | Negative | 0.208 | 0.693 |
| 1:512 |  | Negative | 0.181 | 0.602 |
| 1:1024 |  | Negative | 0.180 | 0.601 |
| 1:2048 |  | Negative | 0.169 | 0.562 |
| 1:4096 |  | Negative | 0.161 | 0.537 |
| 1:8192 |  | Negative | 0.157 | 0.523 |
| 1:16384 |  | Negative | 0.090 | 0.300 |

Representative images of the second-generation Hcp1-ICT results. Red circles indicate the test line. OD_450_ of 0.3 corresponds to 1 ELISA Unit/mL (EU/mL).
