# Supplementary material for: Development and international multicenter evaluation of a second-generation immunochromatography test for the serological diagnosis of melioidosis
Source: PLoS Negl Trop Dis. 2026 Jul 6;20(7):e0014484. doi: 10.1371/journal.pntd.0014484 (PMC13379097; doi:10.1371/journal.pntd.0014484)
Supplement: S3 Table — (DOCX) [file pntd.0014484.s003.docx]

**S3 Table** Clinical and serological characteristics of culture-confirmed melioidosis patients from the Australian cohorts included in the evaluation of the second-generation of Hcp1-ICT.

| Site | No. of patients | Median IHA titer | IHA ≤ 1:20  n (%) | Serum collected ≤ 3 days before or on the day of melioidosis diagnosis, n (%) | Serum collected > 3 days before of melioidosis diagnosis, n (%) | Hcp1-ICT positive  n (%) |
| --- | --- | --- | --- | --- | --- | --- |
| Darwin | 16 | < 1:20 | 14 (87.5) | 11 (68.8) | 3 (18.8) | 3 (18.8) |
| Townville | 65 | 1:80 | 24 (36.9) | 14 (21.5) | 3 (4.6) | 36 (55.4) |
