# Supplementary material for: Development and international multicenter evaluation of a second-generation immunochromatography test for the serological diagnosis of melioidosis
Source: PLoS Negl Trop Dis. 2026 Jul 6;20(7):e0014484. doi: 10.1371/journal.pntd.0014484 (PMC13379097; doi:10.1371/journal.pntd.0014484)
Supplement: S4 Table — (DOCX) [file pntd.0014484.s004.docx]

**S4 Table** Specimen sources of B. pseudomallei among culture-confirmed melioidosis patients included in the study.

| Country | No. of patients | Blood culture positive (bacteraemia) n (%) | Non-blood specimens culture positive n (%) |
| --- | --- | --- | --- |
| Thailand | 150 | 103 (68.7) | 47 (31.3) |
| Darwin, Australia | 16 | 9 (56.3) | 7 (43.7) |
| Townsville, Australia | 65 | 65 (100) | 0 (0) |

Bacteraemia was defined as isolation of B. pseudomallei from blood culture. Non-blood specimens included sputum, pus, urine, pleural fluid, synovial fluid, peritoneal dialysis fluid, and other clinical specimens.
